# Maternal Interaction With Infants Among Women at Elevated Risk for Postpartum Depression

**DOI:** 10.3389/fpsyg.2022.737513

**Published:** 2022-03-03

**Authors:** Sherryl H. Goodman, Maria Muzik, Diana I. Simeonova, Sharon A. Kidd, Margaret Tresch Owen, Bruce Cooper, Christine Y. Kim, Katherine L. Rosenblum, Sandra J. Weiss

**Affiliations:** ^1^Department of Psychology, Emory University, Atlanta, GA, United States; ^2^Department of Psychiatry, University of Michigan, Ann Arbor, MI, United States; ^3^Department of Obstetrics and Gynecology, University of Michigan, Ann Arbor, MI, United States; ^4^Department of Community Health Systems, University of California, San Francisco, San Francisco, CA, United States; ^5^Department of Psychology, The University of Texas at Dallas, Richardson, TX, United States; ^6^The Pennsylvania State University (PSU), University Park, PA, United States

**Keywords:** postpartum, depression, infant, interaction, maternal

## Abstract

Ample research links mothers’ postpartum depression (PPD) to adverse interactions with their infants. However, most studies relied on general population samples, whereas a substantial number of women are at elevated depression risk. The purpose of this study was to describe mothers’ interactions with their 6- and 12-month-old infants among women at elevated risk, although with a range of symptom severity. We also identified higher-order factors that best characterized the interactions and tested longitudinal consistency of these factors from 6 to 12 months of infant age. We leveraged data from eight projects across the United States (*n* = 647), using standardized depression measures and an adaptation of the NICHD Mother-Infant Interaction Scales. Overall, these depression-vulnerable mothers showed high levels of sensitivity and positive regard and low levels of intrusiveness, detachment, and negative regard with their infants. Factor analyses of maternal behaviors identified two overarching factors—“positive engagement” and “negative intrusiveness” that were comparable at 6 and 12 months of infant age. Mothers’ ability to regulate depressed mood was a key behavior that defined “positive engagement” in factor loadings. An exceptionally strong loading of intrusiveness on the second factor suggested its central importance for women at elevated depression risk. Mothers with severe depressive symptoms had significantly more “negative intrusiveness” and less “positive engagement” with their 6-month-old infants than women with moderate or fewer depressive symptoms, suggesting a potential tipping point at which symptoms may interfere with the quality of care. Results provide the foundation for further research into predictors and moderators of women’s interactions with their infant among women at elevated risk for PPD. They also indicate a need for evidence-based interventions that can support more severely depressed women in providing optimal care.

## Introduction

Postpartum depression (PPD) occurs in approximately 13% of women (Bauman et al., 2020) and is associated (concurrently and prospectively) with adverse psychological and developmental functioning in children (Stein et al., 2014; Weiss and Leung, 2021). Efforts to understand why or how PPD is linked to offspring functioning suggest the salience of interactions between mothers and their infants in mediating outcomes (Murray et al., 2015; Jones et al., 2019; Goodman et al., 2020; Pelham et al., 2021). The qualities of mothers’ interactions with their infants can be reliably and validly measured with observational coding or rating (Tryphonopoulos et al., 2016; Bornstein et al., 2020). Elevated depressive symptom levels have been associated with maternal interactions that are more negative or coercive, less positive, and more disengaged (Lovejoy et al., 2000; Hakanen et al., 2019), albeit not all mothers with elevated depression exhibit such behaviors (Goodman et al., 2017; Weiss and Leung, 2021). Yet, it is unclear whether these qualities of parenting generalize to mothers who are at elevated risk for PPD but do not necessarily have PPD or experience high symptom levels. Better understanding the nature of maternal interactions among these women can enhance our ability to target our interventions more effectively and prevent potentially adverse outcomes for children. Thus, we aimed to identify higher-order factors that best characterize maternal behaviors of women at risk for PPD when interacting with their 6-month-old infants and to test the longitudinal consistency of these factors from 6 to 12 months of infant age.

Population-based research shows that, among women in general, risk factors for PPD are highly prevalent, and their presence may be more the norm than the exception (Norhayati et al., 2015; Zhao and Zhang, 2020; Ahmad et al., 2021; Liu et al., 2021). Recent reviews of research to date have identified primary risks for PPD. These include the following: a previous history of depression and anxiety (including depression during pregnancy), negative attitudes toward the pregnancy, premenstrual syndrome, stressful life events (including parenting stress), a history of abuse, current domestic violence, perinatal complications, and a high-risk pregnancy, having a low birth weight or prematurely born infant, hormonal dysregulation, young age, socioeconomic disadvantage, sleep dysfunction, nutritional deficiencies, lack of exercise, lack of social support, acculturation, and marital or partner dissatisfaction (Ghaedrahmati et al., 2017; Chen et al., 2019; Hutchens and Kearney, 2020). From among these many factors, we focus on three in this study: (1) history of depression, including bipolar depression (Bukh et al., 2016; Mandelli et al., 2016; Silverman et al., 2017; Buckman et al., 2018), (2) having a medically high-risk pregnancy or a preterm, low birth weight infant (Carson et al., 2015; Hawes et al., 2016; Shlomi Polachek et al., 2016; de Paula Eduardo et al., 2019), and (3) a history of childhood maltreatment (Hutchens et al., 2017; Humphreys et al., 2020; Perry et al., 2020; Weiss et al., 2021). We were able to leverage data from women representing these important risk populations from previously funded studies of our research group. Availability of these data presented an important opportunity to expand knowledge about these risks. Sampling such populations, relative to general population samples, may yield a more ecologically valid representation of populations experiencing PPD.

A focus on women at elevated risk for PPD is important for three reasons. First are the high rates of occurrence in the population for these particular risk groups. Among US women, approximately 22% experience depression in contrast to 15% of men (Centers for Disease Control and Prevention (CDC), 2020). The lifetime prevalence of delivering a preterm, low birth weight infant is on average 12%, but with substantially higher percentages among women of specific racial/ethnic groups (Schaaf et al., 2013; Frey and Klebanoff, 2016; Keiser et al., 2019). The prevalence of various types of childhood maltreatment is also substantial, including 20% for sexual abuse, 22% for physical abuse, and 28.4% for emotional abuse among women in North America (Stoltenborgh et al., 2015; Moody et al., 2018).

Second, each of these elevated risks for PPD pose risks for adverse outcomes for the offspring. Depression in women during the perinatal period is reliably associated with elevated risk for the development of a broad range of adverse outcomes in offspring, beginning during infancy (Goodman and Lusby, 2014) and throughout development (Stein et al., 2014). Research indicates that children of mothers with bipolar depression are at particular risk of psychopathology and varied developmental problems (Lau et al., 2018), including mood and sleeping disorders (Sandstrom et al., 2019; Wescott et al., 2019). Among women with a preterm, low birth weight infant, PPD has also been linked to the child’s emotional and behavioral problems (Gueron-Sela et al., 2015; Kleine et al., 2021), including depressive and anxiety disorders (Weiss and Leung, 2021). Similarly, PPD among women who experience trauma has been associated with their child’s increased risk of emotional and behavioral problems (Choi et al., 2017). Plant et al. (2017) found that the effects of PPD in traumatized women carried forward into early adolescence of the offspring, with maladaptive parenting playing a mediating role.

A third reason to focus on women at elevated risk for depression stems from previous studies of interactions with their infants. Women’s history of depression is associated with their providing less optimal qualities of interaction with their young children, even when symptom-free (Kluczniok et al., 2016) while mothers’ bipolar depression is associated with less attunement and less dyadic coordination (Anke et al., 2019). A history of child maltreatment is linked to less sensitivity toward infants by mothers (Fuchs et al., 2017), and having a medically fragile infant (such as those born prematurely) is related to more intrusive behavior as well as greater remoteness and negative affect when mothers are depressed (Neri et al., 2015). Findings from these studies raise concern about maternal interactions among these groups of women who are at elevated risk for PPD. However, many of the previous studies are of small samples and examine each risk factor in isolation, despite knowledge that risk factors co-occur (Goodman and Tully, 2009). There is a lack of knowledge about maternal behavior that may cross-over risk factors and be more generally representative of women at elevated risk. Moreover, researchers often rely on idiosyncratic approaches to measuring mothers’ qualities of interacting with their infant, giving less attention to higher-order constructs that may characterize interactions with the infant across high-risk populations.

### Aims of the Study

In the study described here, we address these shortcomings by examining mothers’ interactions with infants among a relatively large sample that includes women with a range of symptom severity and across a spectrum of these risks for PPD. In addition, we extend studies of individual parenting characteristics that have used a well-established measure of mother–infant interaction by identifying overarching constructs from interaction qualities in the measure that are salient for mothers at elevated depression risk. Our specific aims were as follows: (1) to describe mothers’ interaction with their infants among a group of women at elevated risk for PPD, including qualities of interaction particularly salient to depression; (2) to identify higher-order factors that best characterize maternal behaviors of women at risk for PPD when interacting with their 6-month-old infants, (3) and to test the longitudinal consistency of these factors from 6 to 12 months of infant age. Although our primary focus was to identify overarching constructs that reflect maternal interactions in high-risk groups regardless of symptom severity, we also examined differences in these interactions based on level of depression severity.

## Materials and Methods

### Design and Procedures

To address our aims, we leveraged and merged data from eight samples of women at diverse risk for PPD. These samples were derived from a network of researchers affiliated with the National Network of Depression Centers, each of whom had obtained video-recorded observations of mother–infant interaction as part of previously funded research with women at heightened risk for PPD. The eight samples were from three universities in different geographic regions of the United States (California, Georgia, Michigan). Three of the projects were conducted at X University, three at University of XX, and two at University of XXX. All women in these projects ranged from 18 to 45 years of age. X University: In two projects, women were recruited during pregnancy from hospital and community clinics and had a history of a primary diagnosis of Major Depressive Disorder. In the 3rd project, women were recruited through a Women’s Health Program in the Department of Psychiatry and Behavioral Sciences and had a history of Bipolar I or Bipolar II Disorder. All women and their infants were followed from pregnancy to 12 months postnatal, collecting data at various time points. University of XX: All women from projects at XX had either delivered a preterm infant or were at risk of preterm delivery when enrolled in the study. Women in two XX projects were recruited from neonatal intensive care units in three major teaching hospitals while women in the 3rd project were recruited from obstetric clinics affiliated with XX, the Public Health Department or the city’s General Hospital. One study followed women and their infants from recruitment during the 1st week of life through 6 months postnatal. In a second study, they were followed from birth to 2 years of age, and in the last study, women were followed from the 3rd trimester of pregnancy to 12 months postnatal. Data were collected at various time points throughout the projects. University of XXX: In one study, women were recruited either during pregnancy or within the first 4 months postpartum and had a history of childhood maltreatment (sexual, physical, and emotional abuse or neglect). In the second project, they were enrolled in the first 12 weeks of pregnancy and had a history of Major Depressive Disorder. Women were recruited either from three prenatal care clinics or through community advertisement. Women and their infants were followed from enrollment to 14 or 18 months postpartum (depending on the project), collecting data at various time points.

Although the eight research projects recruited women at different time points to address the specific goals of their original funding, all had data on women’s depression and interactions with their infants at 6 months postpartum. Each project used (a) questionnaires and interviews to measure sample characteristics and depression, and (b) video records to capture mothers’ interactions (*n* = 647 at infant age 6 months; *n* = 346 at 12 months). Six out of the eight projects collected data at 12 months of infant age, allowing for a test of consistency (internal validity) of the parenting constructs that would be derived from the 6 months data. Institutional Review Boards at the three universities approved the research projects.

### Participant Recruitment and Inclusion Criteria

All projects had similar recruitment approaches, with participants being recruited either during pregnancy or in the early postpartum at obstetric and/or neonatal units or through advertisements. Further, all participants were at elevated risk of depression, with risk defined as women with either: (a) a history of major depressive disorder or bipolar disorder; (b) a history of childhood maltreatment; or (c) delivery of a preterm or low birth weight infant.

### Measurement of Depressive Symptoms

Each project measured mothers’ depression symptom severity at infants’ ages 6 and 12 months with either: the Beck Depression Inventory (Beck et al., 1988, 1997), the Postpartum Depression Screening Scale (Beck and Gable, 2000), or the Patient Health Questionnaire-9 (Kroenke et al., 2001). Each of these measures has established validity and reliability, including for perinatal populations. We harmonized depression data by using validated cut-points for each measure that have been determined previously by researchers who developed the measures (Beck et al., 1997; Beck and Gable, 2001; Kroenke et al., 2001). Based on these cut-points, we combined women across projects into comparable depression severity groups (i.e., none or minimal, mild, moderate, and severe) at each of the two infant ages. An ordinal variable was created for each cut-point (e.g., 1 = none or minimal depression and 2 = mild depression) that allowed us to group women across projects who met criteria for each category when performing our analyses.

### Mothers’ Interaction With Infants

#### Observational Procedures

In all of our study projects, mothers’ interaction was observed during an unstructured free-play procedure, with the same basic instructions to mothers to “please play with your baby in a way that is typical for you.” These similarities reduced the likelihood of confounding effects from different interactional scenarios. In order to standardize timing of the ratings, we followed a practice, common in the field, of rating the first 5 minutes of directed play interaction for all videos, regardless of duration (e.g., Hirsh-Pasek et al., 2015). In their meta-analytic review of mothers’ depression in relation to parent–child interaction qualities, Lovejoy et al. (2000) found weak and inconsistent evidence that interaction duration was a moderator. Further supporting the use of 5-min observational samples, a large longitudinal study reported evidence for construct validity of a latent parenting construct derived from observational ratings of a 5-min teaching task (Nordahl et al., 2020).

Although the context for assessment of mother interaction had many similarities across the studies, protocols of the different projects varied in whether observations occurred at home or in a lab. However, we examined the effects of the setting in which observations were recorded as well as the effect of project site and found that they did not influence mothers’ interaction. In tests of between-subjects effects using a general linear model, partial eta squared tests for effect size of project site (a, b, c) ranged from 0.000 to 0.004 (at *p* = 0.34 or greater); $\eta_{p}^{2}$ for effect size of setting (lab versus home) ranged from 0.002 to 0.003, at *p* = 0.19 or greater.

#### Interaction Rating System

We used a well-regarded rating system from the NICHD Study of Early Child Care and Youth Development (SECCYD) to analyze qualities of mothers’ interaction from video records (see NICHD Early Child Care Research Network, 1999). Although the original NICHD SECCYD mother–infant interaction rating system also included infant rating items and one dyadic item, we used only the parent items in the study reported here. In addition, we added two items from the Parent-Child Early Relational Assessment (Clark, 1985) to enhance salience of the rating system for comorbid mood problems common in interactions of women at risk of depression. These items included mothers’ *Depressed/Withdrawn/Apathetic Mood and Anxious Mood*. Our final measure consisted of nine items: *Sensitivity/Responsiveness to Distress, Sensitivity/Responsiveness to Nondistress, Intrusiveness, Detachment/Disengagement, Stimulation of Development, Positive Regard for the Child, Negative Regard for the Child, Depressed/Withdrawn/Apathetic Mood, and Anxious Mood*.

We used 5-point versions of the rating scales, from 1 (*not at all characteristic*) to 5 (*highly characteristic*), which were expansions of the original 4-item version (Owen, 2006). Several large-scale longitudinal studies have used the 5-point and/or a 7-point version (Mills-Koonce et al., 2007; Nordahl et al., 2020). This rating system yields strong evidence for reliability and validity, including inter-rater reliability estimates (Fuligni and Brooks-Gunn, 2013). Predictive validity includes support from the NICHD SECCYD and other work (e.g., Campbell et al., 2004; Booth-LaForce and Oxford, 2008; Norcross et al., 2017). Overall, the rating system has shown a high level of validity as a measure of mother–infant relationship quality.

The two items included from the Parent-Child Early Relational Assessment are also rated on a 5-point Likert-type scale (PCERA; Clark, 1999). The PCERA has good discriminant validity, distinguishing between parents based on various definitions of high risk (e.g., psychiatric diagnoses), and is sensitive to therapeutic intervention change (Clark, 1999; Clark et al., 2008; Unternaehrer et al., 2019). In factor analytic work, *depressed/withdrawn/apathetic mood* (reverse scored) loaded on a factor of Parental Positive Affective Involvement and Verbalization; *anxious mood* loaded on a factor of Parental Intrusiveness, Insensitivity, and Inconsistency (Clark, 1999).

#### Rating Processes and Determining Reliability of Observers

At each site, at least two research assistants completed the ratings. We followed recommended procedures in training observers to obtain and maintain reliability (Bakeman and Goodman, 2019). We took several steps to achieve the criterion we set for reliability: observers differing by no more than 1 point on no more than 4 items for ratings of 4 consecutive videos relative to “master ratings” of an expert who had been responsible for the centralized coding of parent–child interactions in the NICHD SECCYD. Once observers met this criterion, they independently rated all the videos with 6-month-olds. Prior to rating the videos with 12-month-olds, observers participated in a brief training on developmental norms for this age and discussed how observations might differ for various items from mothers’ interactions with 6-month-old infants. We required observers to meet the criterion for reliability for coding videos of 12-month-old infants, based on our expert’s “master ratings” of 12-month-olds.

We determined inter-observer reliability by having at least 20% of randomly selected video segments at each infant age rated by a second trained observer. Across sites, observers had adequate agreement as determined by being within 1 point on each rating scale, that is, greater than 0.80 on all items. They had agreement between 0.90 and 1.00 on most items. All sites used one REDCap database to enter ratings as well as information on the context within which the interaction occurred and the video quality. Using a double data entry system for accuracy, there were errors of less than 1% across sites.

### Approaches to Data Analysis

To examine Aim 1, we used descriptive statistics to compute sample means for the rating items at each age. For Aim 2, we used two methods to identify factors that might reflect underlying constructs that could characterize maternal interaction behavior. First, we estimated exploratory factor analyses (EFA) to determine how specific rating items might group together to form latent variables. We computed the EFA with the item scores from mothers’ interactions with their 6-month-old infants. Then, we performed a confirmatory factor analysis (CFA) to determine how well the factor structure identified at 6 months fit the items rated when infants were 12 months old. This latter measurement model addressed our aim of testing the longitudinal consistency of these factors from 6 to 12 months of infant age (Aim 3).

Because the rating scales were short Likert-type scales and many of their distributions were skewed, they did not meet the necessary assumptions for ordinary least squares analyses using product–moment correlations. Thus, we used an EFA using robust maximum likelihood (MLR) with a probit link for both 6- and 12-month items, treating indicators as ordinal. We carried out the EFAs for both ages using GEOMIN oblique rotation to assist in achieving a solution with simple structure, while still allowing factors to be correlated. In this approach, we expected each factor to have item loadings of 0.40 or larger and expected items to load strongly on a single factor. Given the number of items, we examined from one- to three-factor solutions only. In addition to evaluating the pattern matrices estimated with the oblique rotation, we examined the scree plot of eigenvalues. We computed the CFA at 12 months to establish that the factors at 12 months had support for metric invariance when compared with those from the 6-month solution. Further, we examined the 6- and 12-month factor structures in the same model, with MLR and a probit link, using numerical integration with Monte Carlo estimation. This analysis employed full information maximum likelihood (FIML) with the expectation–maximization algorithm to accommodate missing data at 12 months.

Lastly, we employed general linear models to examine differences in mean scores on the factors we identified across women who had varying levels of depression severity at each infant age. We carried out statistical analyses with Stata 15 and Mplus. We evaluated all tests of significance with a two-sided alpha of 0.05.

## Results

### Participant Characteristics

Women were, on average, 31.3 years old (±5.9). 87.5% were married or lived with a partner. Most women had a bachelor’s degree or some graduate education (63%). 66% reported their racial and ethnic heritage as White/European American, 15% were Black/African American, 10% were Hispanic American/Latina, 6% were Asian American, and 3% reported other or mixed race/ethnicity. 17% received government assistance and were below the poverty level. A third of the women reported minimal satisfaction with the support they were receiving. In addition, 21% met the threshold for clinical anxiety and 33% reported a history of physical or sexual abuse. Infants’ average gestational age at birth was 36.7 weeks (±4.1); 30% were born preterm. Male to female split among infants was 50:50. Further details can be found in Table 1.

**Table 1:** Characteristics of the sample.

Variables	
Maternal sociodemographic characteristics	*N* (%) or *M* (*SD*)
Maternal age Mean (SD)	31.3 (5.9)
Highest level of education Less than high school (HS) HS Graduate or GED Some college/vocational/other Post-HS Bachelor’s degree Post-graduate	50 (7.7) 49 (7.5) 136 (21.1) 195 (30.1) 217 (33.6)
Race/Ethnicity White/European American Black/African American Hispanic/Latina Asian American More than One Race	427 (66.1) 97 (15.0) 64 (9.8) 37 (5.7) 22 (3.4)
Marital status Married/living with partner Single/Separated/Divorced/Widowed	566 (87.5) 81 (12.5)
Below poverty level/relied on government assistance No Yes	535 (82.7) 112 (17.3)
Perceived social support^*^ Low satisfaction Moderate satisfaction High satisfaction	220 (34) 207 (32) 220 (34)
**Maternal clinical status and history**	
Depression Minimal or no depression Mild Moderate Severe	378 (58.4) 124 (19.2) 95 (14.7) 50 (7.7)
Meets threshold for clinical anxiety No Yes	459 (70.9) 188 (29.1)
Substance abuse during pregnancy No Yes	628 (97.1) 19 (2.9)
History of childhood physical or sexual abuse No Yes	434 (67.1) 212 (32.9)
**Infant characteristics**	
Sex Male Female	323 (49.9) 324 (50.1)
Apgar Score < 7 No Yes	586 (90.6) 61 (9.4)
Birthweight in grams Mean (SD)	2973.1 (952.7)
Gestational age in weeks Mean (SD)	(4.1)
Born prematurely No Yes	453 (70) 194 (30)

* Divided into tertiles.

### Descriptive Statistics: Maternal Depression and Maternal Interactions

At 6 months postpartum, 7.7% (*n* = 50) of the women reported severe depressive symptoms, 14.7% (*n* = 95) reported moderate symptoms, 19.2% (*n* = 124) reported mild depression, and 58% (*n* = 378) had minimal or no depressive symptoms. At 12 months postpartum, 5.8% (*n* = 20) of the women met criteria for severe depression. 7.8% (*n* = 27) of the women reported moderate symptoms, 15% (*n* = 52) reported mild depression, and 71.4% (*n* = 247) endorsed minimal or no depressive symptoms.

Table 2 presents the rating scale item means and standard deviations at each age. Given few instances of infant distress, we eliminated consideration of the item *Sensitivity to Distress*. Item means indicate that observers rated mothers in our sample as being on the higher end for *sensitivity* and *positive regard*, in the moderate range of *cognitive stimulation*, and low on *intrusiveness, detachment, and negative regard*. Table 3 shows bivariate correlations among items. Overall, items had significant relationships with one another. An exception is the item *anxious mood*, which had low correlations with other items at 6 months and non-significant relationships with all but one item (*negative regard*) at 12 months.

**Table 2:** Means and standard deviations (SD) for interaction rating scale^*^ item scores at infant ages 6 and 12 months.

Scale Item	6 Months (*n* = 647)		12 Months (*n* = 346)	
	Mean	*SD*	Mean	*SD*
Sensitivity	3.73	0.94	3.72	0.91
Intrusiveness	1.75	1.04	1.44	0.77
Detachment	1.40	0.75	1.42	0.79
Cognitive Stimulation	2.92	1.03	3.07	1.03
Positive Regard	3.81	0.78	3.69	0.70
Negative Regard	1.24	0.57	1.24	0.51
Depressed Mood	1.18	0.47	1.22	0.52
Anxious Mood	1.11	0.37	1.11	0.37

* NICHD Early Child Care Research Network (1999). Child care and mother-child interaction in the first 3 years of life. Dev. Psychol. 35, 1399–1413.

Clark, R. (1999). The parent-child early relational assessment: a factorial validity study. Educ. Psychol. Measure. 59, 821–846.

**Table 3:** Bivariate spearman correlations for mothers’ interaction items at 6 months (upper triangle) and 12 months (lower triangle) postpartum.

	*S*	*I*	*D*	CS	PR	NR	DM	AM
Sensitivity (S)	−0.46^*^	−0.54^*^	0.45^*^	0.61^*^	−0.28^*^	−0.32^*^	−0.14^*^	
Intrusiveness (I)	−0.39^*^		0.28^*^	0.03	−0.21^*^	0.36^*^	0.15^*^	0.16^*^
Detachment (D)	−0.49^*^	0.13		−0.23^*^	−0.57^*^	0.32^*^	0.52^*^	0.15^*^
Cognitive Stimulation (CS)	0.49^*^	−0.03	−0.23^*^		0.40^*^	−0.04	−0.14^*^	0.05
Positive Regard (PR)	0.54^*^	−0.13	−0.55^*^	0.35^*^		−0.30^*^	−0.48^*^	−0.11^*^
Negative Regard (NR)	−0.35^*^	0.25^*^	0.32^*^	−0.21^*^	−0.34^*^		0.25^*^	0.13^*^
Depressed Mood (DM)	−0.33^*^	0.05	0.50^*^	−0.25^*^	−0.48^*^	0.23^*^		0.15^*^
Anxious Mood (AM)	−0.13	0.08	0.09	−0.07	−0.02	0.18^*^	0.08	

* Correlations are significant at *p* < 0.01.

### Exploratory Factor Analysis: Identifying Factors That Best Characterize Interaction of Women With Their 6-Month-Old Infants

**Figure 1:**
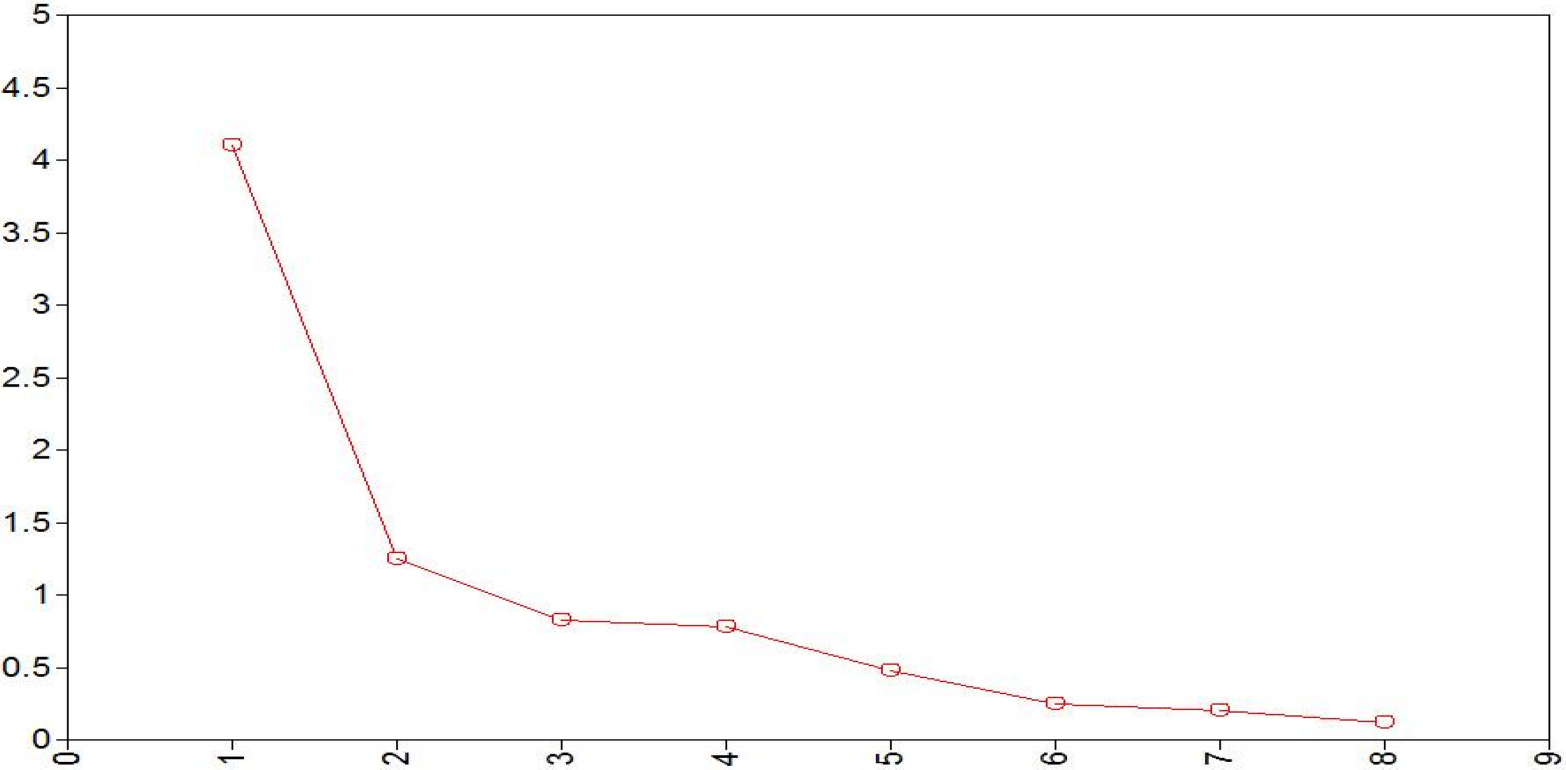
Scree plot with eigenvalues of factors from exploratory factor analysis at 6 months.

A scree plot for initial EFA on items rating mothers’ interaction with their 6-month-olds (see Figure 1) indicates that the eigenvalue for one of the factors was quite strong (*λ* = 4.10), explaining much of the variance in the rating items. However, a second factor also exceeded an eigenvalue of 1 (*λ* = 1.25), with one being the accepted minimal criterion for a factor to be useful in explaining the variance contributed to a construct by at least two items in the measure. Based on these findings, we examined both a one- and two-factor solution in our EFA.

In the EFA for the one-factor solution, *anxious mood* did not meet the criterion of 0.40 to be retained in a well-defined factor. The Bayesian Information Criterion (BIC) was 9241.22. A two-factor solution using geomin oblique rotation improved the BIC to 9082.23. In addition, a chi-square difference of 320.50 (*df* = 7, *p* = 0.000) between models indicated that the 2-factor solution significantly improved the fit. The item *anxious mood* again did not load on either factor (loadings were −0.23 and 0.24, respectively). In light of this finding, we computed the final EFA for the 6-month ratings of maternal behavior without *anxious mood.* Loadings for the final two-factor solution are shown in Table 4.

**Table 4:** Final factor loadings for two-factor solution of scale items reflecting maternal behaviors at 6 and 12 months postpartum.

	6 Months		12 Months	
	Factor 1	Factor 2	Factor 1	Factor 2
Sensitivity	**0.717**	−0.279	**0.674**	**−0.382**
Intrusiveness	0.000	**1.032**	0.001	**0.998**
Detachment	**−0.853**	0.089	**−0.846**	0.006
Cognitive Stimulation	**0.547**	0.186	**0.553**	0.022
Positive Regard	**0.928**	0.043	**0.847**	0.009
Negative Regard	−0.375	**0.413**	**−0.429**	**0.355**
Depressed Mood	**−0.757**	0.001	**−0.794**	−0.115

All bolded items are significant at *p* < 0.05.

Using the established criterion of 0.40 as a desired loading for a well-defined factor, Factor 1 consisted of five items: *positive regard*, *sensitivity*, *cognitive stimulation*, *detachment* (negatively loaded), and *depressed/withdrawn/apathetic mood* (negatively loaded). We defined this factor as *Positive Engagement*. Factor 2 consisted of two items: *intrusiveness* and *negative regard*, which we labeled *Negative Intrusiveness*. The two factors were moderately intercorrelated, *r* = −0.39, *p* = 0.0000.

### Confirmatory Factor Analysis: Testing the Longitudinal Consistency of the Factors

Factor loadings for a 2-factor structure of mothers’ interactions with their 12-month-old infants are shown in Table 4, again using goemin oblique rotation. Items loaded in a similar pattern to that found for the 6-month data, with *sensitivity, detachment, cognitive stimulation, positive regard,* and *depressed mood* all clearly loading on one factor while *intrusiveness* loaded on another. The only difference between the 6- and 12-month loadings was for *negative regard*. As with the 6-month data, it cross-loaded on both factors. However, the strength of its loading was slightly stronger on Factor 1 than on Factor 2 at 12 months. When we estimated factor structure for both 6- and 12-month data in the same model (using FIML with the probit link and an EMA algorithm), the pattern of coefficients was consistent with our prior analyses, supporting a cross-loading of *negative regard* on both factors and its slightly stronger loading on Factor 1 than Factor 2 at 12 months. Finally, we estimated an additional model in which the factor loadings were constrained to be the same for each item. For example, the factor loading for the item *positive regard* was constrained to be equal on the first factor for the 6-month data and the 12-month data. In comparing the BIC of the constrained model (12949.62) to that of the unconstrained model (12991.72), the constrained model showed a significant improvement in fit by 50 points. This provides evidence that the constrained model fits the data better than a model with factor loadings free to vary, supporting metric invariance of the 6- and 12-month factor solutions.

### Severity of Depressive Symptoms and Maternal Interaction

Table 5 shows data on differences for factor scores of women who had varied degrees of symptom severity at both infant ages. With 6-month-old infants, women with severe depressive symptoms differed significantly on both factor scores from women in all other symptom groups. Women classified as having severe depressive symptoms were significantly lower on Factor 1, *F* = 4.04 (*df* = 3), *p* = 0.007, and significantly higher on Factor 2, *F* = 7.48 (*df* = 3), *p* = 0.000. With their 12-month-old infants, maternal symptom severity was not associated with scores on either factor. Analyses of variance yielded *F* = 0.92 (*df* = 3), *p* = 0.43 for Factor 1 and *F* = 1.98 (*df* = 3), *p* = 0.12 for Factor 2.

**Table 5:** Factor scores for women with different degrees of depression severity at 6 and 12 months postpartum.

	6 Months				12 Months			
	Factor 1^*^		Factor 2^**^		Factor 1		Factor 2	
	*M*	*SD*	*M*	*SD*	*M*	*SD*	*M*	*SD*
Minimal/No Depression	3.21	0.56	1.40	0.63	3.13	0.59	1.31	0.47
Mild Depression	3.21	0.64	1.47	0.58	3.18	0.67	1.36	0.55
Moderate Depression	3.23	0.57	1.62	0.76	3.32	0.60	1.39	0.61
Severe Depression	2.91	0.77	1.82	0.89	3.13	0.47	1.60	0.82

* *F* = 4.04 (*df* = 3), *p* = 0.007;

** *F* = 7.48 (*df* = 3), *p* = 0.000.

Neither factor at 12 months showed significant differences between groups.

## Discussion

Understanding interaction with infants among women with PPD is important given evidence that qualities of interaction may mediate associations between PPD and offspring’s development of psychopathology (Goodman et al., 2020). However, research efforts to understand mother–infant interaction in women with PPD may be misinformed if they do not study women at elevated risk for PPD who have a range of symptoms, including women who present with minimal or no symptoms in spite of elevated risk. To address this important gap in knowledge, we took advantage of a consortium of researchers affiliated with the National Network of Depression Centers, harmonizing data from eight projects that had enrolled women at elevated risk for PPD and had video records of mother–infant interactions.

As a result of their elevated risk, the prevalence and severity of mothers’ depression in our sample were higher than is typically reported for the general population, whether with convenience- or population-based sampling. Approximately 22% of women reported moderate to severe depressive symptoms (indicative of clinically significant depression) when their infant was 6 months old, declining to about 14% when the infant was 12 months of age. Putnick et al. (2020) identified varied trajectories of PPD symptoms over time in a large, population-based birth cohort: (1) high, persistent symptoms, (2) low stable symptoms, (3) initially low but increasing symptoms over time, and (4) initially moderate symptoms that decreased over time. They found the largest group of women to have persistently low stable symptoms. As a whole, women in our sample appeared to reflect a trajectory involving mild to moderate symptoms that decreased from 6 to 12 months postpartum. However, we did observe a subgroup of about 7.7% of women in our sample who had more severe symptoms that appeared to persist through 12 months.

### Interactions With Infants Among Women at Elevated Risk of Depression

Overall, mothers’ interaction with their infants showed relatively high levels of sensitivity and positive regard, moderate levels of developmental stimulation, and low intrusiveness, detachment, and depressed or anxious mood. Our descriptive finding that mothers’ interactions were relatively high on positive qualities and relatively low on negative qualities may be explained in several ways. First, although our samples of mothers were all at high risk for PPD, they were, on average, low in sociodemographic risk. Being relatively well-resourced may enhance positive parenting directly or indirectly, protecting against negative effects of PPD on mother–infant interaction (e.g., Martinez-Torteya et al., 2014). Second, this pattern of findings is consistent with other research demonstrating that not all mothers at risk for PPD have poor quality interaction with their infants (e.g., Goodman et al., 2017; Weiss and Leung, 2021). Interactions with infants are not determined by any single factor, such as risk for PPD, but by multiple factors, including the mother’s personal psychological resources, children’s characteristics, and contextual factors (Belsky and Jaffee, 2006). Third, women with mild to moderate depressed states, the majority of our sample, may be more able to readily engage in positive interactions with their infants (Pechtel et al., 2013). This hypothesis was supported by our finding that more severely depressed mothers, relative to mothers with lower symptom levels, had less positive engagement and more negative intrusiveness when interacting with their infants.

In contrast to our findings showing relatively high positive qualities and relatively low negative qualities of maternal interaction, the women in our sample provided, on average, only a moderate degree of cognitive stimulation with their infants. We found that cognitive stimulation, characterized by engagement in activities that can facilitate learning, such as focusing the infant’s attention on perceptual qualities (sounds, colors, movement), was significantly, inversely associated with ratings of mothers on depressed mood during their interactions with infants. This is consistent with Hakanen et al.’s (2019) finding that mothers’ depressive symptoms were associated with lower structuring when interacting with their 6-month-old infants. These findings are also consistent with other studies showing that non-postpartum adult populations with depression show cognitive impairment even when in remission (Listunova et al., 2018). Although we demonstrated a link between reduced cognitive stimulation and depressed mood, we need to interpret findings for the entire sample with caution since women in our study experienced a spectrum of depression, from no symptoms to severe symptoms.

### Key Overarching Factors in Mothers’ Interactions

From among the individual qualities of maternal interaction just described, we found support for two overarching factors. One represented positive behavior (higher sensitivity, positive regard and cognitive stimulation, and lower detachment and depressed mood), and the other represented negative behavior (intrusiveness and negative regard). Given that the strongest loadings on our first factor were for a low degree of detachment and a high degree of positive regard, we named it *Positive Engagement*. We named our second-factor *Negative Intrusiveness*. Although the strength of specific items and the resulting names given the factors vary, our identification of two key factors (*Positive Engagement* and *Negative Intrusiveness*) is congruent with findings from general population samples that have used the NICHD SECCYD rating system (Mills-Koonce et al., 2007; Burchinal et al., 2008; Hibel et al., 2011; Willoughby et al., 2013).

Our *Positive Engagement* factor has similar items to the *Positive Sensitivity* factor in these previous studies, consisting of sensitivity/responsiveness, detachment [reverse scored], positive regard, animation, and stimulation of development. However, positive regard and a strong degree of engagement with the infant were the items carrying the strongest weights in our first factor, rather than sensitivity *per se*. Also, our unique inclusion of the item on *depressed mood* enabled us to show that mothers’ ability to regulate or manage any depressed affect/behavior in their interactions with infants may be a key component of the *Positive Engagement* factor. With women at elevated risk for PPD, this *depressed mood* item may be particularly informative, tapping qualities such as blunted or restricted affect and apathetic, listless behavior when interacting with the infant. The item, *animation* (energy level), included in some studies of general populations, may assess one dimension of depressed affect and behavior, but not its affective complexity.

Our second factor, *Negative Intrusiveness*, in line with Hibel et al. (2011) conceptualization, provides strong support for intrusiveness and negative regard as behaviors constituting a second higher-order construct in mother–infant interactions among women at risk for PPD. For our sample of women at elevated risk, the strong factor loadings for intrusiveness at both 6 and 12 months indicate its central importance as a construct within this population, in contrast to some studies of community samples where intrusiveness did not load on any factor (Mills-Koonce et al., 2015; Nordahl et al., 2020).

The rating item *anxious mood* did not load significantly on either of the two factors we identified. This finding is somewhat surprising in light of the recognized comorbidity of depression and anxiety, including in postpartum women (Goodman and Tully, 2009; Flynn et al., 2018; Weiss et al., 2021) and the related potential for their synchronous effects on interaction. However, we observed generally low levels of anxious behavior at both time points. We hypothesize that women’s potential comorbid anxiety may have presented itself through other interaction items, both in our first factor where disengagement was a central maternal behavior and in our second factor of *negative intrusiveness*. Further study is needed to understand the role of observed anxiety in mother–infant interactions among women at high risk for PPD.

### Longitudinal Consistency of Mothers’ Interactions

Our factor analyses showed overall support for the same two factors for mothers’ interactions with their 6- and 12-month-old infants. The maternal behavior of *negative regard* was the only one that seemed questionable. It cross-loaded on the factors at both time points but loaded a bit more strongly on the *negative intrusiveness* factor at 6 months and on the *positive engagement* factor (reverse scored) at 12 months. However, the loadings were both significant and their strength was very similar. Conceptually, *negative regard* is more congruent with the *negative intrusiveness* factor, as we more clearly found in the 6-month factor solution. The pattern of coefficients across factor solutions at 6 and 12 months of infant age as well as their valences were very consistent across the two time points.

### Depression Severity and Mothers’ Interactions

Our results showed significantly higher levels of negative intrusiveness and lower levels of positive regard with 6-month-old infants among women with concurrent severe depressive symptoms than for women with moderate or fewer depressive symptoms. These findings are consistent with previous research (Slomian et al., 2019) and suggest a certain threshold or tipping point at which mothers have more difficulty managing their symptoms during interactions with their infants, ultimately interfering with the quality of their care. It is noteworthy, however, that the potential effect of more severe depressive symptoms on mothers’ interaction was no longer evident with the 12-month-old infants. Mothers who are more severely depressed may develop improved coping strategies over time, a hypothesis needing examination in future research. Alternatively, although the proportion of women with severe symptoms did not change from 6 months (7.6%) to 12 months (7.4%), our decreased overall sample size at 12 months may have reduced the power to detect significant differences observed at 6 months of infant age.

### Limitations and Strengths

Overall, our findings should be interpreted within the context of certain limitations. First, although about a third of the sample was not White, most women were married or cohabiting, and college educated. Only 17% were living in poverty. In addition, we recognize that the risk groups we studied are not the only ones at risk of depression. As noted in the introduction, there are many other populations of women at risk of PPD who may have unique mother–infant interactions, including women with minimal or no social support, those with significant socioeconomic disadvantage, or women who are immigrants and have high levels of acculturative stress. Thus, findings may not generalize to women at elevated risk of depression for other reasons. Second, mean levels of observed depressed and anxious mood during mothers’ interactions with infants were low, even in these samples of women at high risk for PPD. Nevertheless, approximately 42% of the women had mild, moderate, or severe depression symptoms. Future studies might consider observing maternal behavior in situations other than free play (e.g., involving stressor paradigms), which may be more challenging to women at risk for PPD, and, thus, more likely to elicit signs of depressed or anxious mood. Third, our use of video recordings of interactions that were collected for previous studies resulted in varied conditions under which interactions were recorded. However, as noted earlier, we examined the effects of the context in which recordings were made as well as the effect of project site and found that they did not influence mothers’ interaction at either 6 or 12 months postpartum. In addition, Lovejoy et al.’s (2000) meta-analytic review found that type of observation (whether structured vs. unstructured or lab vs. home) does not significantly moderate the association between mothers’ depression severity and their interaction with infants.

Despite its limitations, the study has numerous strengths. We leveraged data and video records already available from previous research, applying a common metric and standardized approach. The merging and standardization of our data enabled a much larger and more diverse sample for examining our aims than is typically available in a single study. In addition, we relied on widely used and well-established rating scale items. One of the rating scale’s original authors served as the core trainer for this research and her ratings were used as the “gold standard” for interactions evaluated in the training and reliability process. Another strength was our broad definition of “elevated risk for PPD,” which enhances the external validity of our findings. Finally, we were able to test longitudinal consistency by studying interactions at two infant ages, 6 and 12 months.

### Research Implications

Our findings suggest important areas for further research. First, there is a need to determine whether the maternal interactions we identified among our sample of women will generalize to women from other groups of elevated depression risk (e.g., mothers of infants with congenital disorders or women with varied medical problems). Second, we found that cognitive stimulation of infants was quite limited in this sample of mothers and that it was inversely related to depressed mood. More in depth research is needed to understand how mothers’ depressed mood may undermine the ability to enhance their infant’s cognitive development, including both biological and psychological mechanisms. Lastly, identification of factors that may moderate the relationship between more severe depression and mothers’ interactions with infants is critical. Such moderators could represent potential targets for early intervention to prevent adverse effects.

Our findings support the value of screening mothers’ interactions with their infants in ongoing clinical care, using psychometrically sound measures (Weiss and Quides, 2012; Zalewski et al., 2017; Fagan et al., 2019). Such assessments could have profound utility in preventing later developmental and mental health problems for children. Interventions could then be designed that integrate prevention or treatment of women’s depression with enhanced parenting skills, directed especially to women at elevated risk for PPD and the ultimate goal of healthy child development (Muzik et al., 2009; Goodman et al., 2017).

## Data Availability Statement

The raw data supporting the conclusions of this article will be made available by the authors, without undue reservation.

## Ethics Statement

The studies involving human participants were reviewed and approved by University of California, San Francisco; University of Michigan; and Emory University. The participants provided their written informed consent to participate in this study.

## Author Contributions

SG, MM, and SW designed the study and had oversight of all study components at their respective Universities. SG, SK, CK, and MO had responsibility for procedures related to coding of video-records. BC, SK, and SW were responsible for harmonizing all study data and for statistical analyses to examine research aims. All authors contributed to interpretation of the findings, writing, reviewing and approval of the manuscript.

## Funding

This research was funded by NICHD, R01 HD084813 [MPIs: SW (Contact), SG, MM]. Other supports to the research include the following: NIMH (MH080147, PI: MM); the Michigan Institute for Clinical and Health Research (UL1TR000433, PI: MM); NIMH (MH065062, PI: Vazquez); NIMH University of Michigan GCRC (M01 RR00042); NICHD (R01 HD081188, PI: SW); NINR (R01 NR002698, PI: SW); Robert C. and Delphine Wentland Eschbach Endowment (PI: SW); NINR (T32 NR016920, PI: SW), NIMH (1P50MH58922-01A1, PI: Nemeroff); NIMH (1 P50 MH077928-01A1, PI: Stowe), NIMH (K23MH096042, PI: DS); Brain & Behavior Research Foundation (PI: DS).

## Conflict of Interest

The authors declare that the research was conducted in the absence of any commercial or financial relationships that could be construed as a potential conflict of interest.

## Publisher’s Note

All claims expressed in this article are solely those of the authors and do not necessarily represent those of their affiliated organizations, or those of the publisher, the editors and the reviewers. Any product that may be evaluated in this article, or claim that may be made by its manufacturer, is not guaranteed or endorsed by the publisher.
